# Metabolomic profiling of exosomes reveals age-related changes in ovarian follicular fluid

**DOI:** 10.1186/s40001-023-01586-6

**Published:** 2024-01-03

**Authors:** Yanqiong Gu, Xunyi Zhang, Ruixue Wang, Yingying Wei, Hao Peng, Kai Wang, Han Li, Yazhong Ji

**Affiliations:** 1 Clinical and Translational Research Center, Shanghai Key Laboratory of Maternal-Fetal Medicine, Shanghai Institute of Maternal-Fetal Medicine and Gynecologic Oncology, Shanghai First Maternity and Infant Hospital, School of Medicine, Tongji University, Shanghai, No. 2699, West Gaoke Road, Shanghai, 201204 China; 2 Reproductive Medicine Center, Tongji Hospital Affiliated to Tongji University, Shanghai, , No. 389 Xincun Road, Shanghai, 200065 China

**Keywords:** Follicular fluid, Exosome, Metabolomics, Advanced age, Ovary

## Abstract

**Background:**

Female fertility declines with increased maternal age, and this decline is even more rapid after the age of 35 years. Follicular fluid (FF) is a crucial microenvironment that plays a significant role in the development of oocytes, permits intercellular communication, and provides the oocytes with nutrition. Exosomes have emerged as being important cell communication mediators that are linked to age-related physiological and pathological conditions. However, the metabolomic profiling of FF derived exosomes from advanced age females are still lacking.

**Methods:**

The individuals who were involved in this study were separated into two different groups: young age with a normal ovarian reserve and advanced age. The samples were analysed by using gas chromatography–time of flight mass spectrometry (GC–TOFMS) analysis. The altered metabolites were analysed by using Kyoto Encyclopedia of Genes and Genomes (KEGG) analysis to identify the functions and pathways that were involved.

**Results:**

Our data showed that metabolites in exosomes from FF were different between women of young age and women of advanced age. The set of 17 FF exosomal metabolites (*P* ≤ 0.05) may be biomarkers to differentiate between the two groups. Most of these differentially expressed metabolites in FF were closely involved in the regulation of oocyte number and hormone levels.

**Conclusions:**

In this study, we identified differences in the metabolites of exosomes from FF between women of young age and women of advanced age. These different metabolites were tightly related to oocyte count and hormone levels. Importantly, these findings elucidate the metabolites of the FF exosomes and provide a better understanding of the nutritional profiles of the follicles with age.

## Background

Female fertility declines with increased maternal age, and it particularly declines more rapidly after the age of 35 years, with a more considerable decline being observed at an age of 45–50 years when menopause normally occurs [1, 2]. Compared with women who get pregnant at a young age, women over the age of 35 years have a higher risk of pregnancy complications [3, 4]. Previous studies have suggested that the age-related decline in female fertility may be related to a decrease in oocyte quality and granulosa cell dysfunction [5, 6]. However, the process of ovarian aging remains largely unexplored. Follicular fluid (FF) is composed of both the transfer fluid of blood plasma constituents that cross the blood follicular barrier and of the secretory active substance of granulosa and theca cells [7, 8]. It contains hormones, proteins, amino acids, enzymes, fatty acids, cytokines, anticoagulant factors, and antiapoptotic factors [7, 9]. Moreover, FF provides an important microenvironment and nutrition for oocyte development and permits intercellular communication and maturity inside of the follicle [10–12]. The FF composition reflects changes in granular layer secretion and the inner membrane, as well as changes in plasma composition caused by physiological or pathological processes [13].

Extracellular vesicles (EVs) have emerged as being important mediators for communication between cells and organs. EVs are classified into exosomes, microvesicles, and apoptotic bodies (according to their different size, biogenesis, and function) [14]. Exosomes are nanosized membrane vesicles (30–150 nm in diameter) [15] that contain a variety of signalling molecules, such as mRNAs, microRNAs, proteins, and metabolites [16]. The transfer of these components among different cells can have an impact on biological activities [16, 17]. Exosomes have been linked to age-related pathological conditions and a dysfunctional mitochondrial–lysosomal axis [18]. Additionally, senescent cell-derived EVs may act as pro-gerontic factors [19, 20]. Increasing evidence indicates that EVs play important roles in the aging process [21]. Importantly, in exosome formation, cytosolic small molecules (metabolites), including sugars, amino acids, nucleotides, different enzymatic cofactors, and redox regulatory molecules, are also involved in these small vesicles [22–24]. EVs can act as independent metabolic units, with potential effects on the physiology of their microenvironment [25]. Metabolomics may be a powerful tool for discovering the underlying biological processes of aging [26].

Exosomes can be isolated from follicular, uterine, and oviductal fluids [27–30]. Recent studies have suggested that exosomes in FF may carry miRNAs for steroidogenesis and follicular development [17, 31, 32]. Through a comprehensive analysis of the composition of FF exosomes, there exists a promising opportunity to identify specific biomarkers and additional information about the ovarian state. However, as part of the exosome cargo, metabolites have not received enough attention thus far. In this study, we aimed to identify differences in the metabolites of exosomes from FF between women of young age and women of advanced age.

## Materials and methods

### Characteristics of patients

All of the females underwent in vitro fertilization (IVF) or intracytoplasmic sperm injection (ICSI) at Shanghai First Maternity and Infant Hospital from February 2022 to December 2022. Women with medical disorders (such as polycystic ovary syndrome [PCOS], endometriosis, cancer, or a history of ovarian surgery), that could affect follicular development were excluded from the study. Participants were divided into two groups: a young control group (age < 35, *n* = 30) and an advanced age group (age ≥ 40, *n* = 30).

This study was approved by the Scientific and Ethical Committee of the Shanghai First Maternity and Infant Hospital affiliated with Tongji University. All of the participants gave their written informed consent and the collection of samples was approved.

### Exosome isolation

All of the patients received controlled ovarian hyperstimulation by using a combination of gonadotropin-releasing hormone (GnRH) agonist or antagonist and recombinant follicle-stimulating hormone (FSH). FF was collected by using transvaginal ultrasound-guided aspiration from dominant follicles (> 18 mm), (yielding 2–3 ml) at 34 to 36 h after the administration of 10,000 IU human chorionic gonadotropin (hCG). The supernatants were stored at − 80 °C after centrifugation at 3000 ×*g* for 15 min to remove cell debris and other particles. The FF exosomes were collected with an ExoQuick-TC Exosome Precipitation Solution Kit (System Biosciences, Palo Alto, CA), which is widely used to obtain exosomes from different body fluids. However, the obtained exosome sample was not homogenous. In brief, 250 µl of ExoQuick Solution was added to 1 ml of FF in a 1:4 ratio. The mixture was thoroughly mixed and left to incubated overnight at a temperature of 4 °C. After incubation, the mixture was centrifuged at 1500 ×*g* for 30 min at 4 °C and the supernatant was carefully removed. The resulting exosome pellet was then resuspended in 500 µl of PBS and passed through a 0.22 µm filter to obtain the desired solution.

### Transmission electron microscopy

Exosomes were analysed via transmission electron microscopy (TEM) as previously described [33, 34]. Twenty microlitres of exosome suspension (5 µg/µl) was fixed on a continuous grid, after which it was negatively stained with 2% uranyl acetate solution for 1 min and air-dried. The samples were then observed by using an FEI Tecnai G2 spirit transmission electron microscope (FEITM, Hillsboro, OR) at an acceleration voltage of 120 kV [35].

### Nanoparticle tracking analysis

Nanoparticle tracking analysis (NTA) measurements were performed by using a NanoSight NS300 instrument (Malvern Panalytical, Malvern, UK) with a 488-nm laser and sCMOS camera module (Malvern Panalytical). Flow measurements were performed at a flow rate of 50. Three 60-s measurements were taken and the captured data were analysed by using NTA 3.2 software [35].

### Western blot

The concentration of proteins was quantified by using the Pierce BCA Protein Assay Kit (Thermo Fisher Scientific, Waltham, MA) following the manufacturer’s instructions. Proteins were separated via gel electrophoresis on 10% SDS-PAGE gels and then transferred to PVDF membranes via electroblotting [17]. After blocking with 5% BSA, the blots were probed with exosome-specific antibodies (CD9, CD63, CD81, and Hsp70; System Biosciences) at 4 °C overnight. After washing the membranes, they were incubated with secondary antibodies, and proteins were visualized by using enhanced chemiluminescence reagents (Thermo Fisher Scientific) [17].

### Metabolomics analyses

Two internal standards were added to exosomes from 1 ml of FF in each serum sample. Gas chromatography–time of flight mass spectrometry (GC–TOFMS) was used to analyse the samples in the order of "control and aging" by using the electron ionization mode (utilized instrument used: Pegasus HT, Leco Corp., St. Joseph, MI). One quality control (QC) sample and one blank vial were run after each 10 exosome samples. The sample was injected using a splitless mode with a volume of 1 μl. A DB-5 ms capillary column, which was 30 m long and 250 μm in diameter with a 0.25-μm film thickness consisting of 5% diphenyl cross-linked with 95% dimethylpolysiloxane, was used to separate the metabolites. Helium (99.9996%) was used with a constant flow rate of 1 ml/min. The GC oven temperature was initially set to 80 °C and held for 2 min. The temperature was gradually increased from the initial temperature to 180 °C at a rate of 10 °C per minute. It was then further increased to 230 °C at a rate of 6 °C per minute and finally to 295 °C at a rate of 40 °C per minute. The temperature was maintained at a constant level at 295 °C for 8 min. The temperatures for the injection, transfer interface, and ion source were set to 270 °C, 260 °C, and 220 °C, respectively. The mass range was set from 30 to 600 with electron impact ionization of 70 eV. The acquisition rate was 20 spectra per second. The obtained files from the GC–TOFMS analysis were exported in NetCDF format by using ChromaTOF software. (v4.44, Leco Co., Los Angeles, CA) [36]. The CDF files underwent several preprocessing steps, including baseline correction, denoising, smoothing, alignment, time-window splitting, and multivariate curve resolution. These steps were performed by using a toolkit developed by MATLAB 7.0 (The MathWorks Inc. Natick, MA), R 10.2 (Lucent Technologies), and JavaSE 1.6 (Sun Microsystems). Data quality control was ensured by using the internal standard and QC, whereas data normalization was performed by using QC. The ion peaks that were generated by the internal standard were removed to avoid any interference in the final results.

### Identification of metabolic profiles

By using the GC–TOFMS analysis and by comparing mass fragments and recent times (RTs) with the National Institute of Standards and Technology (NIST) 05 and our laboratory libraries, we annotated 127 metabolites. Our libraries encompassed over 800 metabolites and are continuously expanding. A principal component analysis (PCA) score plot based on the samples from the aging group and the normal control group did not exhibit distinct clusters, thus indicating that they were not separated from each other. Partial least-squares-discrimination analysis (PLS-DA) and orthogonal partial least-squares-discrimination analysis (OPLS-DA) models were used to visualize the metabolic differences among the aging and normal control groups. The R2X, R2Y, and Q2Y values of the OPLS model were nearly 1.0 for the aging and normal control groups, thus indicating a good ability to explain and predict variations in the X and Y matrices. The resultant *P* values for all of the metabolites were subsequently adjusted to account for multiple tests by using a false discovery rate (FDR) method. Metabolites with both multivariate and univariate statistical significance (variable importance in projection, VIP > 1 and *P* < 0.05) were considered to be potential markers capable of differentiating PC from the controls. Additionally, the KEGG database was utilized to identify the affected metabolic pathways.

### Data analysis and statistics

Statistical analysis for acquiring data was performed by using SPSS (SPSS, Chicago, IL) and SIMCA-P 12.0.1 + (Umetrics, Umea, Sweden). For the normally distributed continuous variables, Student’s *t* test was used for comparisons between the different groups. Otherwise, the Mann − Whitney *U* test was used. The OPLS models were validated by using a permutation test. Qualitative data were compared by using either the Chi-square test or Fisher's exact test. Spearman’s rank correlation analysis was performed to estimate the correlation between the detected metabolites and the clinical characteristics of all of the subjects, we performed. A statistically significant result was defined as *P* < 0.05.

## Results

### Characteristics of patients

The mean basal serum FSH (mIU/ml) was lower in the young age group than in the advanced age group. The number of retrieved oocytes was significantly higher in the young age group than in the advanced age group. There was no significant difference between the two groups in basal serum luteinizing hormone (LH, mIU/ml) or basal serum estradiol (pg/ml) (Table 1).

**Table 1:** Characteristics of patients

Parameters	Young age group (*n* = 30)	Advanced age group (*n* = 30)	*P*
Age (years)	28.97 ± 0.53	44.13 ± 0.58	< 0.05
BMI (kg/m^2^)	21.35 ± 0.37	22.38 ± 0.37	0.52
Basal serum FSH (mIU/ml)	6.80 ± 0.64	14.58 ± 2.65	< 0.05
Basal serum LH (mIU/ml)	5.65 ± 0.71	5.74 ± 0.89	0.94
Basal serum estradiol (pg/ml)	51.54 ± 6.68	37.53 ± 4.71	0.09
Number of oocytes retrieved	16.5 ± 1.64	3.33 ± 0.72	< 0.05

All of the results are presented as the mean ± SEM

*BMI* body mass index, *FSH* follicle-stimulating hormone, *LH* luteinizing hormone

### Isolation and characterization of exosomes derived from FF of young controls and advanced age

**Fig. 1:**
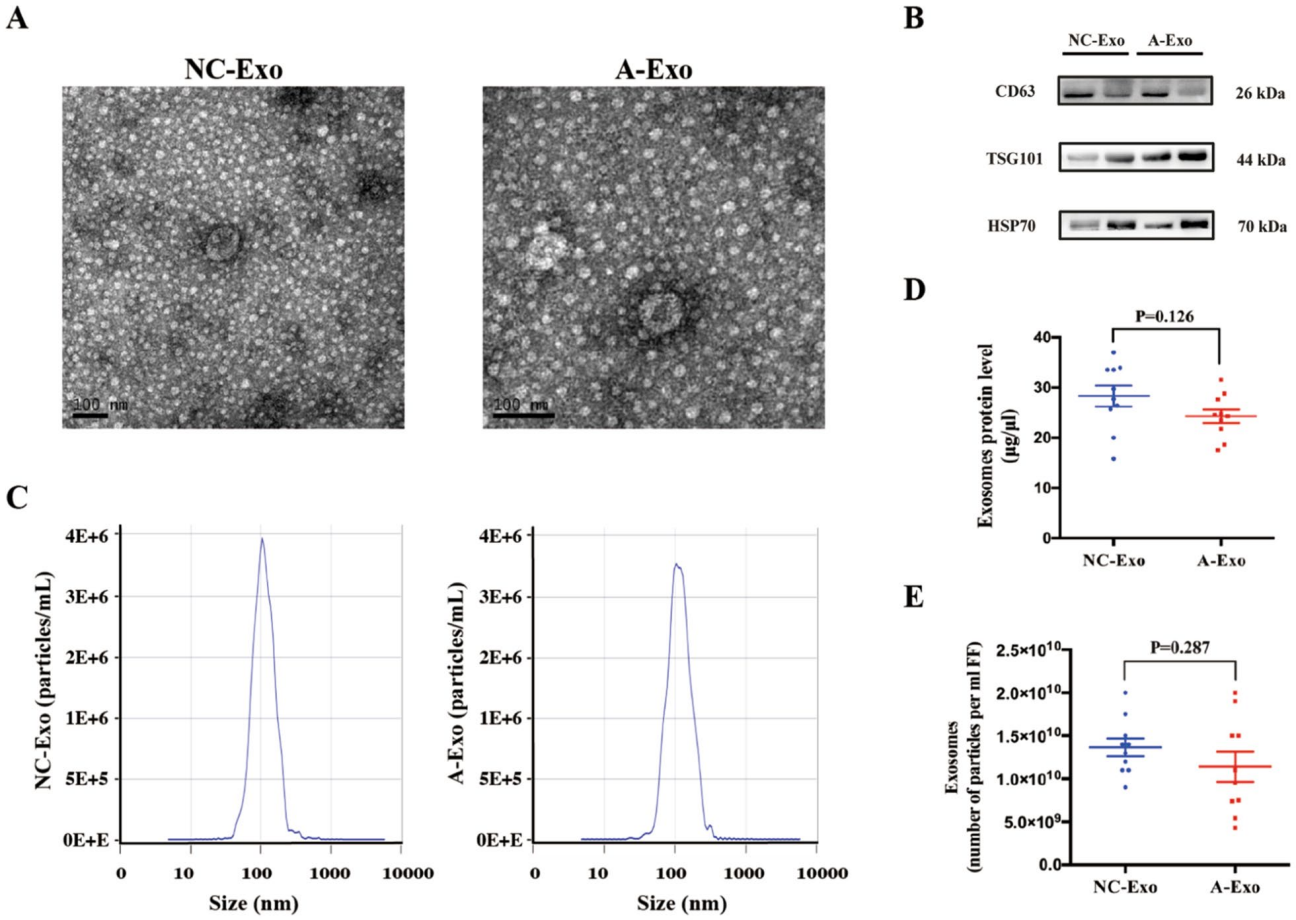
Characterization of exosomes derived from the FF of patients in the advanced age group and young age group. **A** TEM images of isolated exosomes with a saucer‐like shape limited by a lipid bilayer. **B** Western blot analysis of exosomal surface markers CD63, TSG101, and HSP70 in FF‐derived exosomes of the young age group and advanced age group. **C** The size distribution of exosomes obtained from FF of the young age group and advanced age group was analysed by NTA. **D** Exosomal protein concentration (ug exosomal protein/µl FF) in the FF of the young age group and advanced age group. **E** Exosomes concentration (number of particles per ml FF) in the FF of the young age group and advanced age group

Exosomes derived from FF were characterized via Western blot, TEM, and NTA. The NTA and TEM findings were in conjunction with the previously documented traits of exosomes (Fig. 1A and Fig. 1C). The NTA and TEM results were consistent with previously reported characteristics of exosomes [17, 35, 37, 38]. Western blot results showed that three exosomal markers (CD63, TSG101, and HSP70) were detected in exosomes derived from FF (Fig. 1B). In addition, we found that exosome concentration (number of particles per ml FF) and exosomal protein concentration (µg exosomal protein/µl FF) from FF in the advanced age group were slightly lower than those from the young age group; however, the difference was not significant (Fig. 1D, E, all *P* > 0.05).

### Main metabolite groups in FF exosomes

**Fig. 2:**
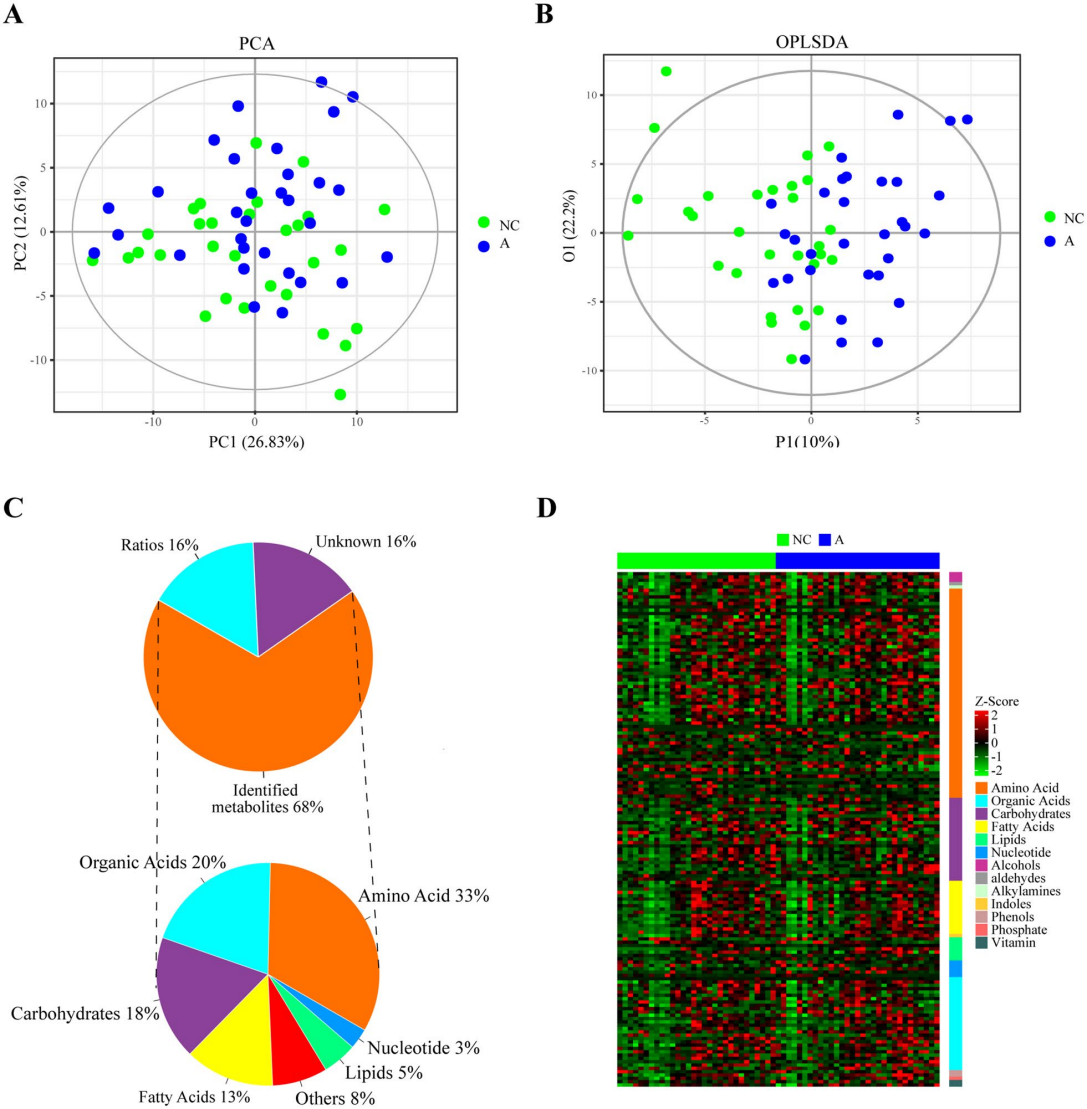
Metabolomic analysis of exosomes derived from FF of the advanced age group and young group. **A** PCA scores of metabolite profiles between the advanced age group and young age group. **B** OPLS-DA model of metabolite profiles between the advanced age group and the young age group. **C** The proportion of identified and unknown metabolites and other components in the sample, as well as the categories and the proportion of identified metabolites in the sample, were also analysed separately. **D** Z score heatmap of an overview of metabolic profiles in all of the samples. The heatmap colour code indicates relative metabolite abundance: red indicates increased levels, and green indicates decreased levels in advanced age vs. young control

Multivariate analysis via principal component analysis (PCA) did not demonstrate a perfect separation between the analysed data groups for each group (PC1 variance: 26.83%; PC2 variance: 12.61%) (Fig. 2A). To further distinguish the differences in metabolic profiles between the two groups, we applied an advanced supervisory discriminant model known as OPLS-DA (Fig. 2B). A clear discrimination between the NC-Exo and A-Exo groups was apparent in the scatter plot of the OPLS-DA model (Fig. 2B). Of all of the samples, identified metabolites accounted for 68%, unknown metabolites accounted for 16%, and other components accounted for 16% (Fig. 2C). The identified metabolites mainly included amino acids (33%), carbohydrates (18%), organic acids (20%), fatty acids (13%), lipids (5%), and nucleotide (3%) (Fig. 2C). To further understand the alterations in metabolites in the two groups, we used a Z score heatmap to provide an overview of the metabolic profile in all of the samples, which showed the relative change in each metabolite across all of the samples (Fig. 2D).

### Age-related changes in the FF exosomal metabolites

We detected 157 metabolites. Of these, 127 metabolites were identified by using *JiaLib™* (one of the largest metabolomics libraries in the world) and 30 metabolites were not identified. Table 2 shows the differentially abundant metabolites that were obtained based on unidimensional statistical analysis in FF exosomes between patients in the young age group and advanced age group, including alcohols, alkylamines, amino acids, carbohydrates, fatty acids, indoles, nucleotides, and organic acids. In particular, we found that the levels of the 13 identified metabolites (one alcohol [2-hydroxypyridine], four amino acids [i.e. ratio of l-glutamic acid/l-glutamine, l-threonine, beta-alanine, and glutathione], two carbohydrates [alpha-lactose, D-maltose], one fatty acid [oleic acid], two nucleotides [inosine and cytidine] and three organic acids [the ratio of homogentisic acid/4-hydroxyphenylpyruvic acid, taurine and fumaric acid]) were significantly higher in the advanced age group than in the young age group, with fold changes ranging from 1.075 to 2.723 (*P* ≤ 0.05). Moreover, four identified metabolites (three amino acids [the ratio of 4-hydroxyphenylpyruvic acid/l-tyrosine, the ratio of l-glutamine/l-glutamic acid and the ratio of ketoleucine/l-leucine], and one organic acid [malonic acid]) were lower in the advanced age group than in the young age group, with fold changes ranging from 0.403 to 0.694 (*P* ≤ 0.05).

**Table 2:** Differences in metabolite expression in FF exosomes between patients in the young age group and advanced age group

Class	Name	*P*	FC
Alcohols	2-Hydroxypyridine	2.00E−02	1.176
Alkylamines	Spermidine	0.072	1.14
Amino acid	Ratio of 4-hydroxyphenylpyruvic acid/l-tyrosine	1.70E−03	0.603
	Ratio of l-glutamine/l-glutamic acid	4.70E−03	0.562
	The ratio of l-glutamic acid/l-glutamine	4.70E−03	1.781
	Ratio of ketoleucine/l-leucine	2.00E−02	0.403
	l-Threonine	2.30E−02	1.326
	Beta-Alanine	2.90E−02	1.301
	Glutathione	3.80E−02	1.378
	l-Arginine	0.052	1.62
	l-Alloisoleucine	0.057	1.124
	Pyroglutamic acid	0.059	1.311
	Ketoleucine	0.082	0.772
	l-Phenylalanine	0.082	1.219
	Aminoadipic acid	0.096	0.72
Carbohydrates	Alpha-Lactose	5.20E−03	1.561
	d-Maltose	7.50E−03	1.833
	l-Arabitol	0.056	1.518
Fatty acid	Oleic acid	3.50E−02	2.668
	Dodecanoic acid	0.08	0.838
Indoles	Melatonin	0.057	1.283
Nucleotide	Inosine	1.30E−02	1.383
	Cytidine	2.90E−02	1.415
Organic acids	Ratio of homogentisic acid/4-Hydroxyphenylpyruvic acid	1.10E−02	2.723
	Taurine	3.60E−02	1.338
	Malonic acid	5.00E−02	0.694
	Fumaric acid	5.00E−02	1.075
	3-Hydroxyanthranilic acid	0.063	1.246
	4-Hydroxyphenylpyruvic acid	0.07	0.578

### Analysis of enrichment pathways between the two groups

**Fig. 3:**
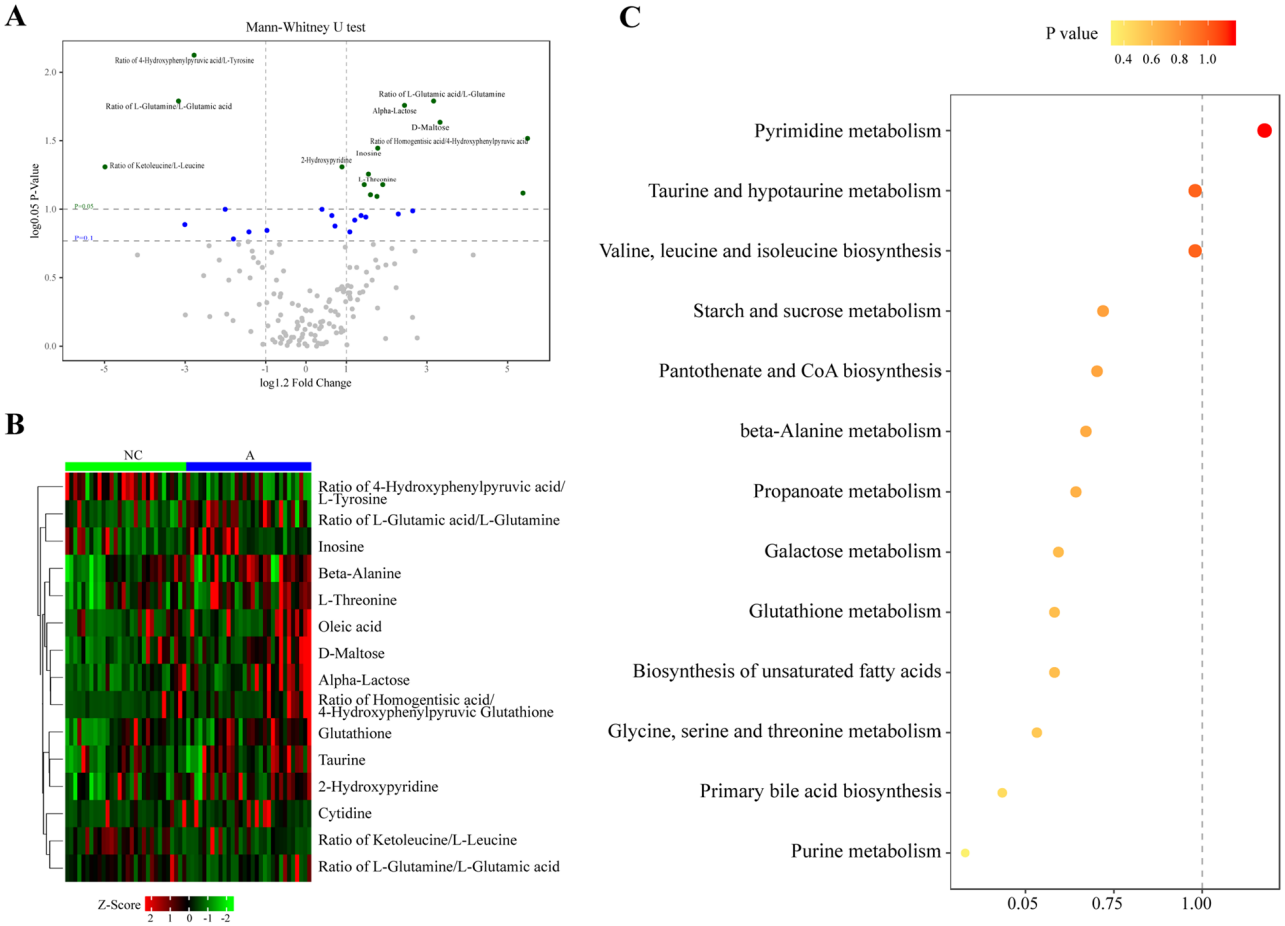
Analysis of differentially abundant metabolites and enrichment pathways between the two groups. **A** Enhanced volcanic map of differentially abundant metabolites based on the one-dimensional criteria (*P* value and fold change value). Levels of each metabolite between the advanced age group and young age group were tested with the Mann–Whitney *U* test. In the volcano plot, the log 0.05 *P* value (y-axis) was plotted against the fold change (x-axis). The metabolites with 0.05 ≤ *P* ≤ 0.1, *P* < 0.05, and *P* ≥ 0.1 are shown with blue, green, and grey points, respectively. **B** Z score heatmap of an overview of differentially abundant metabolites in FF exosomes of the advanced age group and young age group. The heatmap displays the relative metabolite abundance, with red and green indicating increased and decreased levels in the advanced group versus the young group, respectively. **C** Metabolite set enrichment analysis depicted the metabolic pathways in which significant differentially expressed metabolites are involved between the advanced age group and young age group

The enhanced volcano diagram shown in Fig. 3A shows the differentially abundant metabolites that were screened based on the one-dimensional criteria (*P* value and fold change value). In this figure, *P* values set thresholds of 0.05 and 0.1, and log_1.2_ FC (FC is a fold change, multiple of intergroup change) set thresholds of ± 1. As shown in the volcano map based on multidimensional statistics, the highlighted metabolites in the upper right corner were significantly increased in the sample of the advanced age group versus the young age group, whereas the highlighted metabolites in the upper left corner were significantly decreased in the sample of the advanced age group versus the young age group (Fig. 3A). Figure 3B shows a Z score heatmap of significantly differentially abundant metabolites between the two groups. The results were consistent with the differentially abundant metabolite table (Table 2). The pathway analysis that was conducted on differentially abundant metabolites demonstrated significant KEGG pathway enrichment. Differentially expressed metabolites in the pathways were mainly related to pyrimidine metabolism, taurine and hypotaurine metabolism, and valine, leucine, and isoleucine biosynthesis (Fig. 3C).

**Fig. 4:**
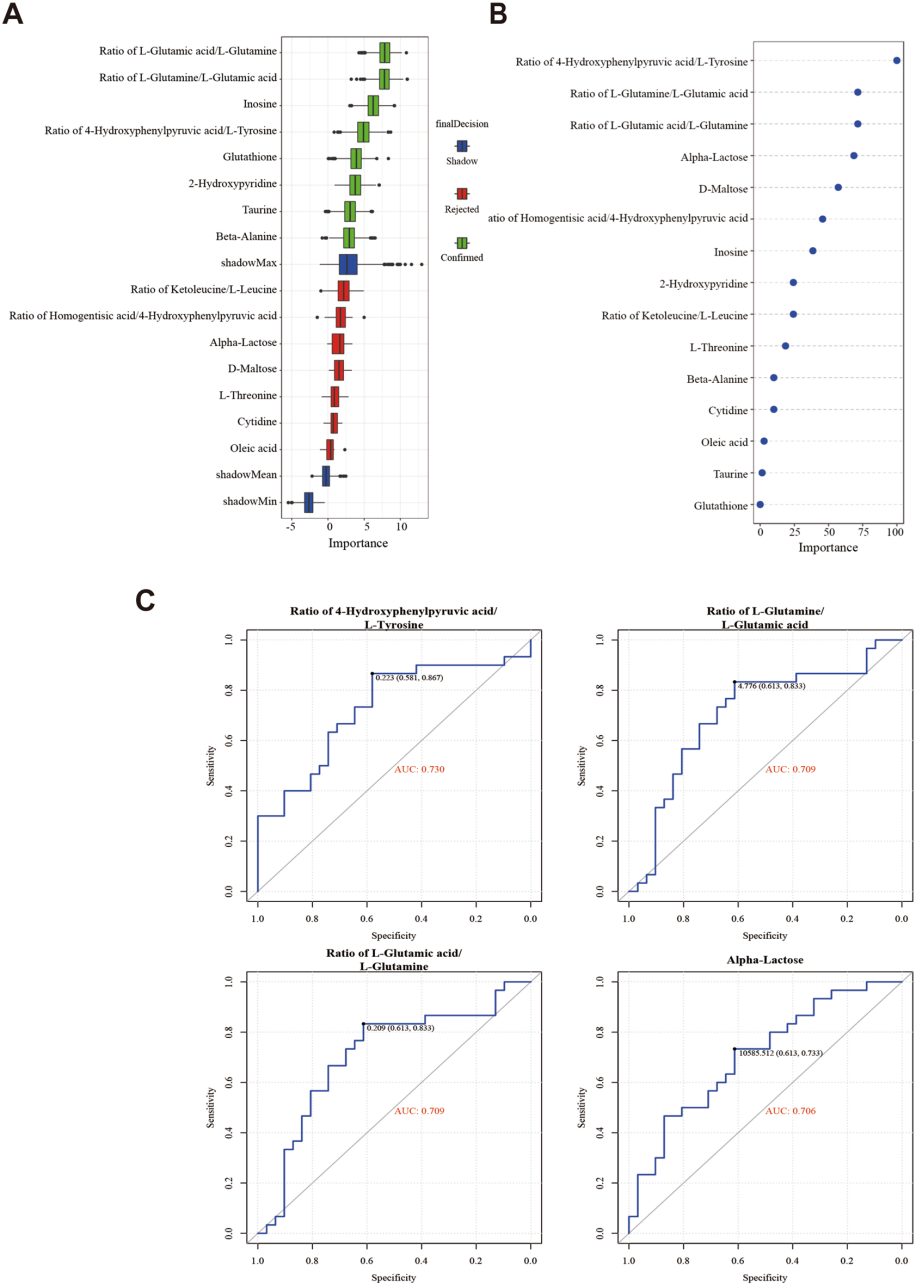
Comparison of the importance of differentially abundant metabolites between the two groups. **A** Random forest assessment of differentially abundant metabolite importance between the two groups. **B** VIP analysis of differentially abundant metabolites between the young age group and advanced age group. **C** Differentially abundant metabolite ROC analysis. The ROC curve of the four metabolites with the largest AUC between the two groups

Given the robust associations of some exosomal metabolites with aging, we, therefore, explored whether exosomal metabolites could serve as biomarkers to differentiate between patients in the advanced age group and the young age group. A total of 29 metabolites were subjected to a random forest classifier for potential metabolite biomarker analyses, and a set of 15 metabolites (the ratio of l-glutamine/l-glutamic acid, the ratio of l-glutamic acid/ l-glutamine, inosine, the ratio of 4-hydroxyphenylpyruvic acid/ l-tyrosine, glutathione, 2-hydroxypyridine, taurine, beta-alanine, the ratio of ketoleucine/ l-leucine, the ratio of homogentisic acid/4-hydroxyphenylpyruvic acid, alpha-lactose, d-maltose, l-threonine, cytidine, and oleic acid) were identified as the optimal set of metabolites to discriminate between the advanced age group and young age group (Fig. 4A). In the variable importance in projection (VIP) variable importance analysis, the differentially abundant metabolite importance between the young age group and the old group was projected (Fig. 4B). We used the 15 metabolites to draw a receiver operating characteristic (ROC) curve, and the area under the curve (AUC) was used as an estimate of the predictive accuracy of the panel of biomarkers. By using the top four important metabolites as indicated by the VIP model (Fig. 4B), an AUC higher than 0.70 was obtained (Fig. 4C). This favourable AUC value demonstrated that these metabolites have a high predictive ability to differentiate patients in the advanced age group from the those in young age group. This result corroborates with the previous models that were built (OPLS-DA), which all performed well in differentiating between the two groups with low error rates. These results suggested that the 15 FF exosomal metabolites may be biomarkers to differentiate between patients in the advanced age group and the young age group.

### Correlation analysis between exosomal metabolites and clinical indicators

**Fig. 5:**
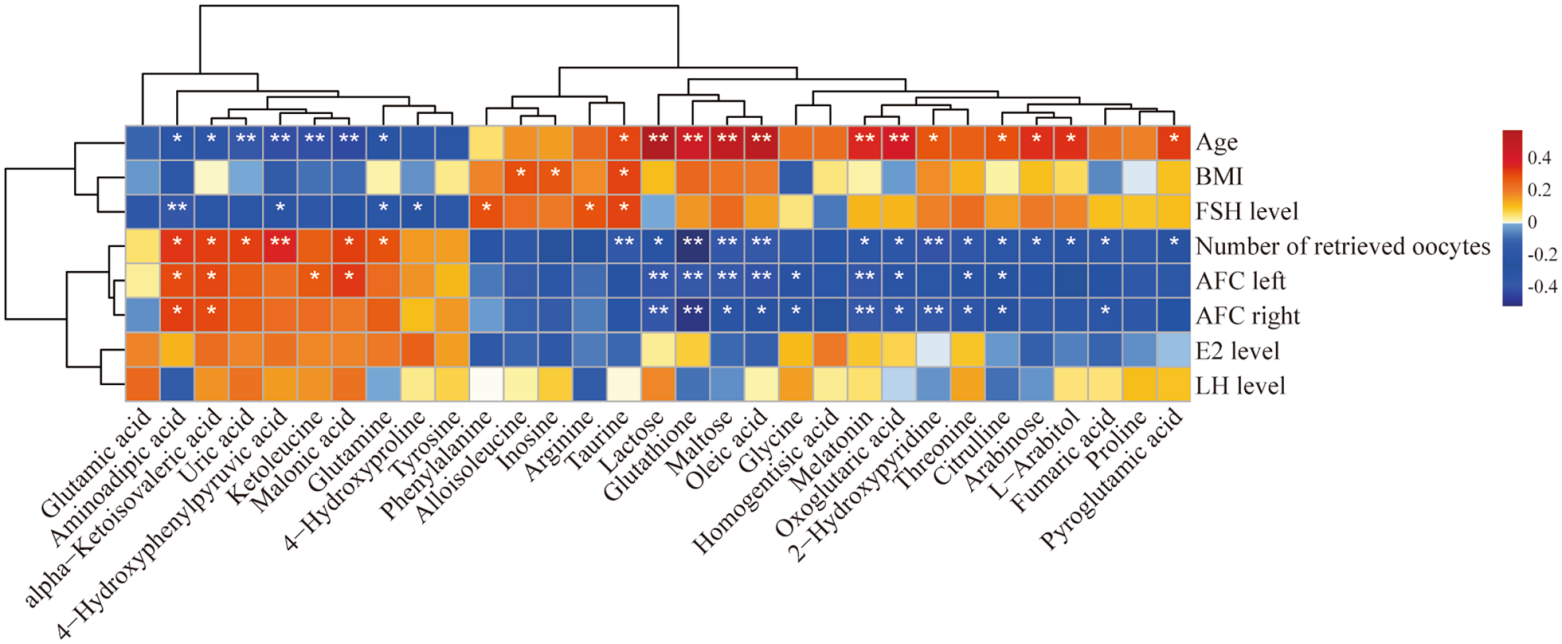
Correlation analysis between exosomal metabolites and clinical indicators. The concentrations of the metabolites from the young age group and advanced age group were normalized according to protein tissue contents. The colours in the heatmap cells indicate correlation; red indicates positive, and blue indicates negative

To analyse the correlation between the detected metabolites and the clinical characteristics of all of the subjects, we performed Spearman’s rank correlation analysis. The overall analysis displayed a clear separation between some clinical characteristics and the concentrations of the metabolites. Among the metabolites that are differentially expressed in FF, some are related to oocyte count and hormone levels (Fig. 5). Several metabolites (i.e. taurine, lactose, glutathione, maltose, oleic acid, melatonin, oxoglutaric acid, 2-hydroxypyridine, citrulline, arabinose, l-arabitol, and pyroglutamic acid) increased with age, and other metabolites (i.e. amino adipic acid, alpha-ketoisovaleric acid, uric acid, hydroxyphenyl pyruvic acid, keto leucine, malonic acid, and glutamine) decreased with age. These results demonstrated an age-related trend in metabolites.

## Discussion

In this study, we observed the metabolic changes in of exosomes in the FF of women of advanced age (which is a natural process) rather than women with premature ovarian failure (POF) or diminished ovarian reserve (DOR). We identified 17 differentially expressed metabolites in the exosomes from FF of young age and women of advanced age (*P* ≤ 0.05, FC > 1 or FC < 1), which were significantly related to the regulation of oocyte number and hormone levels.

Previous studies have reported on the characteristics of FF metabolites rather than exosomes [39]; additionally, other studies have identified the metabolic profile of FF and attempted to find potential biomarkers of oocyte developmental competence [40–43]. Targeted metabolomics has demonstrated a significant reduction in polyunsaturated choline plasmalogens and a lower dimethylarginine/arginine ratio in the FF of patients with diminished ovarian reserve [44]. In addition, analyses of the FF metabolomics profile demonstrated decreased follicular glucose levels and increased lactate levels in advanced maternal-age women, which may suggest that follicular glycolysis is overactive [45, 46]. Unlike these studies, our study focused on metabolites in FF exosomes derived from females of advanced age. Exosomes have a unique composition of inclusions compared to the humoral environment, and the inclusions are more stable because of membrane encapsulation. In the present study, we found that exosome concentration and exosomal protein concentration from FF in the advanced age group were slightly lower than those from the young age group (Fig. 1D, E), which is consistent with a recent study showing that circulating exosome concentration decreases with aging [47]. Additionally, the circulating exosomes of older individuals were more easily internalized by B cells and had higher major histocompatibility complex II (MHC-II) expression on monocytes than exosomes from younger people, thus suggesting that the decreased concentration of exosomes with age may be partially attributed to increased internalization [47].

Our analysis of metabolites in the FF of exosomes showed increased oleic acid (FC = 2.668, *P* < 0.05), alpha-lactose (FC = 1.561, *P* < 0.05), and d-maltose (FC = 1.833, *P* < 0.05) levels in the advanced age group compared with the young age group, which may partly reflect age-related changes in ovarian glycolysis and fatty acid metabolism in older women. Follicles utilize a predominantly glycolytic method of adenosine triphosphate (ATP) production [48]. Oleic acid is a long-chain unsaturated omega-9 fatty acid and is a major monounsaturated fatty acid in the lipid extracts of bovine, sheep, pig [49, 50], and human oocytes [51]. Other studies have found that oleic acid had a positive effect on lipid storage, oocyte maturation, and subsequent embryo development in the cows, thus overcoming the adverse effects of the other two main saturated fatty acids (palmitic and stearic acids) [52]. Several in vitro studies have also indicated that the increased oleic acid concentration in the FF of the advanced age group may reflect decreased lipid storage, insufficient energy storage, and lower cell metabolism.[53–55]. In addition, increased alpha-lactose and d-maltose in the concentration of FF exosomes from the advanced age group may reflect enhanced glycolysis, including increased glycogen decomposition and the conversion of lactose to glucose to provide energy for follicular development [56, 57].

In our results, significantly increased levels of glutathione (FC = 1.378, *P* < 0.05), beta-alanine (FC = 1.301, *P* < 0.05), and l-threonine (FC = 1.326, *P* < 0.05) were identified in the advanced age group. Glutathione exerts antioxidative and free radical-scavenging roles. The concentration of glutathione in oocytes can indicate the stage of oocyte maturation [58, 59]. Studies have found that glutathione levels decline with age [60]. Moreover, the generation of glutathione, which is reversible with antioxidants, is one of the most significant inadequacies of antioxidant defences in aging cells [61]. In our results, significantly increased glutathione in the concentration of exosomes of FF in the advanced age group may indicate increased antioxidant production. In normal metabolism, the ammonium produced by cells is eliminated by the synthesis of alanine, glutamic amide, and the urea cycle. The build-up of ammonium in the FF can lead to metabolic perturbations, alterations in gene expression patterns, and a decline in embryo viability [61]. A likely role for the increased alanine and threonine level in the exosomes of FF in the advanced age group that were observed in this study may be represented by the removal of excess ammonium.

Furthermore, significantly increased levels of inosine (FC = 1.383, *P* < 0.05), cytidine (FC = 1.415, *P* < 0.05), and taurine (FC = 1.338, *P* < 0.05) were observed in the advanced age group. Cytidine plays an important role in phosphoinositide signalling and the synthesis of lipids [62]. In addition to being incorporated into nucleic acids, the circulating pyrimidine known as cytidine can act as a substrate for the salvage pathway of pyrimidine nucleotide synthesis and as a precursor of the cytidine triphosphate (CTP) that is needed in the biosynthesis of phosphatidylcholine and phosphatidylethanolamine [63]. The increased levels of inosine and cytidine in patients of advanced age may reflect the change in nucleotide stress expression and the synthesis of more transfer ribose nucleic acids (tRNAs) and lipids, which support the expression of stress response proteins and energy substrates. The effects of taurine on female reproduction may be primarily achieved by regulating the activities of hypothalamic–pituitary–ovarian axis-related hormones [64]. Increased taurine in the concentration of exosomes of FF in the advanced age group may indicate the compensatory enhancement of ovarian endocrine function with age.

## Conclusions

In this study, we identified differences in the metabolites of exosomes from FF between women of young age and advanced age. These different metabolites have certain significance for revealing the mechanism of age-related changes in ovarian reproductive function. Additionally, these findings may lead to a better understanding of the nutritional profiles of the follicles with age.

## Data Availability

The data that were used in the study are available from the corresponding author upon reasonable request.
